# Causes of Death in HIV-infected Persons Who Have Tuberculosis, Thailand

**DOI:** 10.3201/eid1502.080942

**Published:** 2009-02

**Authors:** Kevin P. Cain, Thanomsak Anekthananon, Channawong Burapat, Somsak Akksilp, Wiroj Mankhatitham, Chawin Srinak, Sriprapa Nateniyom, Wanchai Sattayawuthipong, Theerawit Tasaneeyapan, Jay K. Varma

**Affiliations:** Centers for Disease Control and Prevention, Atlanta, Georgia, USA (K.P. Cain, J.K. Varma); Siriraj Hospital, Mahidol University, Bangkok, Thailand (T. Anekthananon); Thailand Ministry of Public Health, US Centers for Disease Control and Prevention Collaboration, Nonthaburi, Thaliand (C. Burapat, T. Tasaneeyapan, J.K. Varma); Thailand Ministry of Public Health, Nonthaburi (S. Nateniyom); Office of Disease Prevention and Control 7, Ubon Ratchathani, Thailand (S. Akksilp); Bamrasnaradura Institute, Nonthaburi, Thailand (W. Mankhatitham); Bangkok Metropolitan Administration, Bangkok (C. Srinak); Phuket Provincial Public Health Office, Phuket, Thailand (W. Sattayawuthipong)

**Keywords:** HIV, tuberculosis, mortality, cause of death, Thailand, epidemiology, research

## Abstract

Up to 50% of persons with HIV and a diagnosis of tuberculosis (TB) in Thailand die during TB treatment. In a prospective observational study, a team of physicians ascribed the cause of death after reviewing verbal autopsies (interviews of family members about events preceding death), laboratory data, and medical records. Of 849 HIV-infected TB patients enrolled, 142 (17%) died. The cause of death was TB for 38 (27%), including 6 with multidrug-resistant TB and 20 with disseminated TB; an HIV-associated condition other than TB for 50 (35%); and a condition unrelated to TB or HIV for 22 (15%). Twenty-three patients (16%) were judged not to have had TB at all. Death from all causes except those unrelated to TB or HIV was less common in persons receiving antiretroviral therapy (ART). In addition to increasing the use of ART, death rates may be reduced through expanded use of modern TB diagnostic techniques.

## Abstract 2

Many of these patients die of a cause other than tuberculosis; expanded use of antiretroviral therapy and modern diagnostic technologies may reduce case-fatality rates.

Tuberculosis (TB) is one of the most common causes of death among people living with HIV worldwide (*1*). In Southeast Asia, the death rate for HIV-infected TB patients during TB treatment is particularly high, ranging from 20% to 50% (*2**–**5*). HIV-infected patients in Southeast Asia are severely immunocompromised at the time of TB diagnosis, with a median CD4+ T-cell lymphocyte count (CD4) of 54–57 cells/µL (*2**,**6**–**9*). With this degree of immunosuppression, it is likely, but not known, that opportunistic infections other than TB contribute substantially to the high case-fatality rate.

Autopsy studies have helped delineate causes of death among people living with HIV, including HIV-infected TB patients, in sub-Saharan Africa (*10**–**13*). These studies found that the most common causes of death were TB, pneumonia, bacteremia, cerebral toxoplasmosis, and *Pneumocystis jirovecii* pneumonia (PCP). Autopsies are not routinely performed in HIV-infected persons in Asia, and data from Africa may not be generalizable to Asia. Malaria is much less common in Asia than in Africa, and HIV-infected TB patients have more severe immunosuppression and higher death rates than patients in Africa (*2**–**4**,**6**–**9**,**14**–**19*). Understanding actual causes of death may help with identifying effective interventions. In part, on the basis of autopsy studies of HIV-infected patients, programs began providing cotrimoxazole preventive therapy (CPT) to HIV-infected TB patients in Africa. This therapy protects against malaria, PCP, toxoplasmosis, and bacterial pathogens. The reported reduction in death rates from this intervention is large in Africa, but less so in Asia.(*2**,**5**,**16**,**20**–**23*). No published studies have demonstrated an association between CPT and reduced death rates in HIV-infected TB patients in Asia in the era of antiretroviral therapy (ART) (*2**,**16*; US Centers for Disease Control and Prevention [CDC], unpub. data).

Thailand has been greatly affected by the TB/HIV syndemic, i.e., 2 diseases acting synergistically to cause excess illness and death (*24*). Each year, TB develops in 91,000 persons, 15%–20% of whom are HIV-infected (*25**,**26*). To address the TB/HIV syndemic, Thailand recommends regular TB screening for persons with HIV, HIV testing for all TB patients, ART for all people with HIV who have a CD4 cell count <250, and CPT for all TB/HIV patients. Access to HIV treatment has been expanded nationwide. However, in practice, not all patients are treated according to the guidelines (*2*). To understand the causes of death in persons with HIV and a diagnosis of TB and identify possible interventions to reduce death rates, we conducted a prospective, multicenter, observational study of HIV-infected patients being treated for TB in Thailand.

## Methods

### Study Setting and Population

We conducted a cohort study of HIV-infected TB patients at the national infectious diseases referral hospital (Bamrasnaradura Institute) in Nonthaburi province and at public TB treatment facilities in Bangkok, Phuket, and Ubon-Ratchathani provinces. These facilities ranged from outpatient clinics to large public hospitals. There are no known, substantial differences in the HIV or TB epidemics across these 4 provinces. At all sites, treatment for HIV and TB were available from government providers using standardized government-recommended regimens. TB patients were eligible if they were HIV-infected, not pregnant, not incarcerated, >18 years of age, and receiving anti-TB treatment <4 weeks before study enrollment. Patients consenting to study enrollment were followed up from TB treatment initiation to the end of TB treatment. For this study, patients received the usual care for TB, HIV, and other diseases according to physician preference. We did not intervene to modify routine clinical practice. This study was approved by the ethical review committees of the Bangkok Metropolitan Administration, the Thailand Ministry of Public Health, and CDC.

### Data Collection and Laboratory Studies

Patients had 3 study visits: at the beginning of TB treatment, at the end of the intensive phase of TB treatment (usually 2 months after start of treatment), and at the end of TB treatment (usually 6 months after treatment initiation). At the beginning of treatment, patients were interviewed using standardized study forms that asked about demographic characteristics, past and present medical history, knowledge and attitudes related to TB and HIV, and sexual behavior history and drug-use history. At every study visit, patients received a physical examination and provided information about medications taken and any adverse events experienced since their previous visit. Study staff reviewed medical records for any health-related problem that occurred between study visits.

At enrollment, blood samples were tested for liver function enzymes, viral hepatitis, complete blood count, and CD4 count. Sputum and specimens from extrapulmonary sites were collected for acid-fast bacilli smear and for mycobacterial culture, identification, and drug-susceptibility testing.

Although the standard TB treatment regimen in Thailand is 2 months of rifampicin, isoniazid, pyrazinimide, and ethambutol followed by 4 months of rifampin and isoniazid, some providers chose to use nonstandard regimens. Therefore, we categorized regimens used into those likely to be effective and those unlikely to be effective; regimens unlikely to be effective were those for which there were no clinical trials data or international guidelines to support use in HIV-infected TB patients (e.g., a 3-drug regimen of isoniazid, rifampin, and ethambutol) or those that may be appropriate for drug-susceptible TB but were prescribed for patients infected with drug-resistant strains. This classification was done by investigators who were blinded to the patient’s treatment outcome.

### Determination of Cause of Death

For patients who died during TB treatment, study staff obtained death certificates and medical records. Study staff also conducted a verbal autopsy for each patient. Verbal autopsies are a method for assessing causes of deaths, including those related to TB and HIV, in resource-limited countries (*27**–**32*). They involve interviewing a family member or friend who was closely associated with the patient during the period preceding death. These respondents were asked when the patient first became sick, what the symptoms were, how the patient died, and what the respondent believed was the cause of death. At enrollment, patients entering the study consented to allow a family member or friend to be interviewed in the event of their death. Likewise, the respondent also provided informed consent at the time of the interview. For patients who were lost to follow-up or transferred care to a facility not participating in the study, we reviewed the Thai government’s vital status registry to determine whether they had died. Patients who died within 3 months and lost to follow-up or transfer were classified as deaths during study follow-up.

A committee of 3 physicians not involved in care of the patients reviewed the records of all deceased patients, including study forms, verbal autopsy reports, death certificates, medical records, and laboratory data. These 3 physicians reviewed cases together. A decision about the cause of death required agreement of 2 of the 3 physicians. After records review, the committee classified the cause of death as 1 of the following: TB, an HIV-related condition (not TB), or a non-TB/HIV–associated condition. The committee also recorded what it believed to be the specific cause(s) of death and its level of certainty about this determination: uncertain, possible, probable, or highly certain. The level of certainty was based on 3 primary criteria: 1) laboratory, microbiologic, or pathologic evidence of the cause of death; 2) agreement between the physician panel and the cause of death as recorded on the death certificate or medical records; and 3) no other cause that was equally likely to have caused death. High certainty required all 3 of these criteria, probable required 2 of the 3, possible required 1, and uncertain was used for cases that lacked all 3 criteria. For some cases, the committee determined that the patient did not have TB. In these cases, there was no microbiologic evidence of TB, and the patient often had another condition that would explain the symptoms reported. Because TB programs would not have detected that patients undergoing treatment did not have TB, the patients therefore would still be registered. This retention is necessary because results should apply broadly to all HIV-infected persons registered for TB treatment.

### Data Analysis

We described causes of death for all patients who died, stratified by level of certainty (highly certain and probable vs. possible and uncertain). We then compared causes of death for patients who died <60 days after TB diagnosis with those who died >60 days after TB diagnosis. Patients with evidence of disease in multiple sites were classified as having disseminated disease. TB localized to the central nervous system or abdomen was classified as complicated.

For univariate analysis of categorical variables, we compared proportions using the χ^2^ test and, when appropriate, the Fisher exact test. For multivariate analyses of the association between medications used and specific causes of death, we performed a Cox proportional hazards multivariate analysis, after first confirming that the assumptions of the proportional hazards model were met. For this analysis, we excluded patients who died within 14 days after treatment initiation because we presumed these deaths were not preventable through medical treatment.

We created 4 separate models, 1 each for death caused by TB, death caused by an HIV-associated condition, death equally likely to be caused by TB or an HIV-associated condition, and death caused by a non-TB and non-HIV-associated condition. The outcome of interest was death due to the specific cause with any level of certainty (and excluding those who died of other causes), compared with patients who were alive. Outcomes other than death were censored after 1 year of follow-up.

We included data on use of CPT, ART, fluconazole, and an effective TB regimen, and we controlled for CD4. For CPT, ART, and fluconazole. Patients who had taken the medication for at least 14 days before their treatment outcome were categorized as being on the medication, whereas those not on the medication or on it for <14 days were categorized as not being on the medication. We also assessed for confounding according to hospitalization at enrollment, hepatitis C antibody reactivity, abnormal liver enzymes, and type of TB (pulmonary, extrapulmonary without complications, or disseminated/complicated extrapulmonary). We chose to assess for confounding among these variables because they were associated with risk for death in HIV-infected TB patients in Thailand (CDC, unpub. data). We developed our final models by using forward, step-wise variable selection, keeping variables with p<0.05 and those that modified the hazard ratios (HRs) by >10%.

## Results

From May 1, 2005, through September 30, 2007, we enrolled 849 patients, of whom 142 (17%) died during TB treatment. Another 150 (18%) patients either were lost to follow-up or transferred their care to a facility not participating in the study. Among patients who died, the ascribed cause of death was TB for 38 (27%), an HIV-associated condition other than TB for 50 (35%), and a condition not related to TB or HIV for 22 (15%). TB or an HIV-associated condition was equally likely in 32 (23%) patients. Of the 142 patients who died, 23 (16%) were judged not to have had TB at all. Among the 74 patients for whom certainty about the cause of death was probable or highly certain, 29 (39%) died of TB, 33 (45%) died of an HIV-associated condition, and 12 (16%) died of a condition not related to TB or HIV (Table 1).

**Table 1:** Causes of death, stratified by level of certainty, for all enrolled patients who died, Thailand, 2005–2007*

Cause of death	Level of certainty, no. (%) patients		Total no. (%) patients, N = 142
	Probable or high, n = 74	Possible or uncertain, n = 68	
TB	29 (39)	9 (13)	38 (27)
HIV-associated condition (not TB)	33 (45)	17 (25)	50 (35)
TB or HIV-associated equally likely	0	32 (47)	32 (23)
Not TB or HIV-associated	12 (16)	10 (15)	22 (15)

*TB, tuberculosis.

Of the 38 patients who died of TB, 20 (53%) had disseminated TB, including 3 who had disseminated multidrug-resistant (MDR) TB. Including those 3, a total of 6 (16%) had MDR TB. Seven patients had TB involving the central nervous system, including 2 with radiculomyelitis and 1 with MDR TB meningitis (Table 2).

**Table 2:** Causes of death for all enrolled patients who died (N = 142), Thailand, 2005–2007*

Cause of death	No. (%) patients
TB	38 (27)
Disseminated TB (3 with MDR TB)	20 (53)
Central nervous system TB (1 with MDR TB)	7 (18)
Pulmonary TB (2 with MDR TB)	10 (26)
Peritoneal TB	1 (3)
HIV-associated condition	50 (35)
Bacterial infection	6 (12)
Cerebral toxoplasmosis	4 (8)
Disseminated CMV	1 (2)
Fungal infection (other than PCP)	8 (16)
Liver disease	1 (2)
Nontuberculous mycobacteria	10 (20)
PCP	7 (14)
Other infectious cause	5 (10)
Other noninfectious cause	1 (2)
Unknown	7 (14)
TB or HIV-associated condition equally likely	32 (23)
Disseminated mycobacterial disease (TB vs. NTM)	6 (19)
Liver disease	1 (3)
Other infectious cause	3 (9)
Other noninfectious cause	1 (3)
Unknown	21 (66)
Non-TB/HIV–associated condition	22 (15)
Bacterial infection	1 (5)
Liver disease	11 (50)
Stevens-Johnson syndrome	2 (9)
Other infectious cause	1 (5)
Other noninfectious cause	6 (27)
Unknown	1 (5)

*TB, tuberculosis; MDR TB, multidrug-resistant TB; CMV, cytomegalovirus; PCP, *Pneumocystis jirovecii* pneumonia; NTM, nontuberculous mycobacteria.

Patients with HIV-associated deaths had a wide range of diagnoses. Among the 50 patients who died of an HIV-related cause other than TB, 10 (20%) died of nontuberculous mycobacterial infections, 7 (14%) died of PCP, and 8 (16%) died of other fungal infections (including 5 with cryptococcal meningitis) (Table 2). Of the 10 patients whose cause of death was determined to be nontuberculous mycobacteria (NTM), 4 had NTM isolated from a normally sterile site (2 from blood, 1 from bone marrow, 1 from a lymph node). NTM was isolated from sputum in 5 of the remaining patients and stool in the other.

A total of 32 patients died of a condition that was equally likely to be TB- or HIV-related, including 6 (19%) ascribed to disseminated mycobacterial disease. These diagnoses were based on multiple specimens being positive for acid-fast bacilli but no mycobacterial culture confirmation or identification. Finally, 22 patients died of a non-TB, non-HIV–associated condition, including 11 (50%) who died of liver disease (Table 2).

The distribution of causes of death varied when stratified by time from TB treatment initiation. When limited to the 74 patients for whom the cause of death was known with high or probable certainty, 18 (55%) of 33 deaths occurring <60 days after TB treatment initiation were caused by TB, compared with 11 (27%) of 41 deaths occurring >60 days after TB treatment initiation (p = 0.02). Of the 41 persons who died >60 days after initiating TB treatment, 23 (56%) died of an HIV-related condition, compared with 10 (30%) of the 33 patients who died <60 days after TB treatment initiation (p = 0.03).

The median CD4 for all patients enrolled was 55 (interquartile range [IQR] 18–142). Among patients who did not die, the median CD4 was 66 cells/μL (IQR 26–169). Median CD4 was 23 cells/μL (IQR 8.5–96) for persons who died of TB (p<0.01 for comparison with patients who did not die), 18 cells/μL (IQR 8–41) for those who died of an HIV-associated condition other than TB (p <0.01), 18 cells/μL (IQR 4–40) for those in whom TB and an HIV-associated cause of death other than TB were equally likely (p<0.01), and 63 cells/μL (IQR 18–112) among persons who died of a non-TB, non-HIV-associated cause (p = 0.18).

Use of ART, opportunistic infection prophylaxis, and an effective TB regimen, along with other characteristics had varying associations with death due to different causes. Of the 849 patients enrolled in the study, 371 (44%) received ART. Among the 142 patients who died, 36 (25%) received ART; 335/707 (47%) of persons not known to have died received ART. The risk for death caused by TB was lower for persons who took ART (HR 0.2, 95% confidence interval [CI], 0.1–0.5) and higher for patients who were prescribed an ineffective TB regimen (HR 5.0, 95% CI 2.0–12.6) and for those who were hospitalized at enrollment (HR 11.9, 95% CI 4.4–32.1). For death due to HIV-associated causes, ART was associated with decreased risk for death (HR 0.4, 95% CI 0.2–0.7), and being prescribed an ineffective TB regimen was associated with increased risk for death (HR 2.6, 95% CI 1.4–5.1). For patients in whom death due to TB or an HIV-associated cause was equally likely, the risk for death was lower for persons who were prescribed ART (HR 0.04, 95% CI 0.01–0.3) and fluconazole (HR 0.4, 95% CI 0.2–0.98). Decreased CD4 was associated with risk for death in all of these analyses, but use of CPT was not. ART, fluconazole, CPT, ineffective TB treatment, and CD4 were not associated with risk for death from a non-TB, non–HIV-associated cause, but hepatitis C antibody reactivity and abnormal liver enzymes were associated with increased risk for death in this group (Table 3).

**Table 3:** Adjusted hazard ratios for associations between patient characteristics and causes of death among enrolled patients, Thailand, 2005–2007*

Patient characteristic	Death caused by TB, n = 723†	Death caused by non-TB HIV-associated cause, n = 745†	Death caused by TB or HIV equally likely, n = 726†	Death caused by non-TB/HIV condition, n = 719†
Used ART‡	0.2 (0.1–0.5)§	0.4 (0.2–0.7)§	0.04 (0.01–0.3)§	0.9 (0.3–2.6)
Used CPT‡	0.5 (0.1–1.5)	1.0 (0.3–3.2)	1.0 (0.3–3.5)	1.1 (0.2–5.5)
Used fluconazole‡	0.5 (0.2–1.2)	0.8 (0.4–1.6)	0.4 (0.2–0.98)§	1.2 (0.4–3.8)
CD4	0.993 (0.987–0.999)§	0.987 (0.980–0.994)§	0.988 (0.981–0.996)§	1.0 (0.996–1.004)
Ineffective TB regimen¶	5.0 (2.0–12.6)§	2.6 (1.4–5.1)§	0.3 (0.04–2.3)	0.9 (0.2–3.7)
Hepatitis C antibody positive	Not included	Not included	Not included	3.2 (1.2–8.3)§
Hospitalized at enrollment	11.9 (4.4–32.1)§	Not included	Not included	Not included
Abnormal liver enzyme levels#	Not included	Not included	Not included	5.3 (2.2–12.9)§

*TB, tuberculosis; ART, antiretroviral therapy; CPT, cotrimoxazole preventive therapy; CD4, CD4+ T-cell lymphocyte count; Not included, not retained in final model. Patient counts exclude 27 persons who died within 14 days of TB treatment initiation and 13 patients with missing CD4. †Each model includes all patients who survived plus those who died of the specific cause noted (in each category, patients who died of any of the other 3 causes were excluded). Values in parentheses are 95% confidence intervals. ‡Must have been taken for >14 days to qualify as taking medication. §p<0.05. ¶Ineffective regimens were those without supportive clinical trials data, without international guidelines, or not likely to work because of the drug-resistance pattern of the patient’s isolate. #Aspartate transaminase >120 units/L and/or alanine aminotransferase >165 units/L and/or bilirubin >2 mg/dL.

## Discussion

Among HIV-infected persons with a TB diagnosis in Thailand, we found that TB-related deaths were most common within the first 2 months after initiation of TB treatment, but overall, >50% of all deaths occurring during TB treatment were not caused by TB, and some patients actually did not have TB. Multiple interventions are needed, therefore, to reduce death rates in HIV-infected TB patients in Thailand.

Among patients who died of TB, delayed TB diagnosis may be partially responsible.. Of the 38 patients who died of TB, 30 had disseminated TB, MDR-TB, or complicated extrapulmonary TB, conditions that are difficult to diagnose, occur frequently in HIV-infected persons, and have high death rates (*13**,**33**,**34*). Hospitalization at enrollment was strongly associated with increased risk for death caused by TB but not death due to other causes, which further suggests that delay in TB diagnosis may be partially responsible. The World Health Organization recommends that countries with TB/HIV syndemics intensify TB case finding in HIV-infected persons and expand access to TB culture and drug-susceptibility testing (*1**,**35*). If implemented broadly, these strategies could reduce TB-related deaths by diagnosing TB before it is disseminated and severe and by allowing early initiation of second-line TB treatment for drug-resistant TB. Expansion in laboratory capacity and case finding will also need to be coupled to physician training. We found that use of regimens that are not standard or not tailored to the drug-susceptibility pattern of the TB strain was an important risk factor for death.

We also found that using ART during TB treatment was associated with reduced death rates both from TB and from non-TB, HIV-associated conditions. Previous epidemiologic studies in Thailand and other countries have demonstrated marked improvement in duration of survival among HIV-infected TB patients treated with ART during TB treatment (*2**,**7**,**16**,**36**–**38*). Our study confirms this finding and suggests that ART use most likely would dramatically reduce both early and late deaths. Clinical trials are currently attempting to identify the optimum time to initiate ART during TB treatment (*39*).

Among HIV-related causes of death other than TB, the most common causes were NTM disease and fungal infections. For the 4 patients who had NTM isolated from normally sterile sites, NTM most likely was a causative factor, but the role is less clear in those in whom it was isolated from sputum or stool. Expanding mycobacterial culture capacity will help better assess the impact of NTM disease in Asia because NTM may be an underappreciated cause of death among patients clinically diagnosed with TB (*40*). Fungal infections may be preventable with prophylactic antifungal treatment; a previous analysis of risk factors for death in Thailand found that fluconazole was associated with improved duration of survival (*16*). Controlled trials of antifungal prophylaxis may be needed to assess whether it increases survival rates among HIV-infected TB patients in Asia.

Although ART is associated with improved survival and fluconazole may be associated with reduced risk of some causes of death, CPT was not associated with reduced risk for death from any cause. This finding is consistent with that of several other observational studies from Southeast Asia in the era of ART (*2**,**16*; CDC, unpub. data). It is possible that the differing epidemiology of opportunistic infections in the region makes CPT less beneficial or not beneficial at all, or that these studies, none of which were randomized controlled trials, were not adequately controlled or powered to detect a meaningful difference. Randomized controlled trials of the efficacy of CPT in patients receiving ART may be needed in Southeast Asia.

In addition to these specific interventions, which could address the specific causes of death identified in this study, other interventions could decrease the high, early death rates observed in persons with HIV and a diagnoses of TB. First, the median CD4 count among all patients in this study was low. Earlier diagnosis of HIV through regular provider-initiated testing and counseling of TB patients and earlier HIV testing of other persons combined with earlier initiation of ART would result in less immunocompromise and less risk for many of the opportunistic infections found in this population. Next, the impact of TB/HIV can be reduced by prevention of TB in persons with HIV (e.g., improved infection control measures in HIV care settings and use of isoniazid preventive therapy) and prevention of HIV in TB patients through appropriate counseling messages targeting persons with HIV and without HIV.

No method of assessing causes of death is completely reliable, particularly in resource-limited countries where microbiologic testing and postmortem examinations are infrequently performed and many patients die outside of hospitals. We used several imperfect data sources in combination—verbal autopsy, medical record review, death certificate data—to identify the cause of death. Both missed diagnoses and false diagnoses may have skewed our findings, but we could not determine the magnitude and impact of these problems without independent verification of the cause of death. Physicians determining cause of death could have been influenced by their own biases, but our use of a panel of physicians and criteria for ascribing causes should have limited this possibility. Finally, some providers did not always use available microbiologic tests, including blood culture. Failure to use these tests may result in underestimation of some causes of death.

We found that TB-related and HIV-related deaths are likely to be reduced through early initiation of ART and of appropriate anti-TB drug regimens. Expanded use of modern TB diagnostics may also improve survival by diagnosing TB before it is disseminated and severe, identifying drug resistance early, and differentiating between TB, NTM, and other causes of illness. Finally, improvements in general HIV care and treatment, including earlier HIV testing and ART use along with appropriate measures to prevent TB and HIV, should decrease the high early death rates observed.

## References

[1] World Health Organization. Interim policy on collaborative TB/HIV activities. Geneva: The Organization; 2004. WHO/HTM/TB/2004.330 [cited 2008 Jun 22]. Available from http://www.who.int/tb/publications/tbhiv_interim_policy/en/index.html.

[2] Akksilp S, Karnkawinpong O, Wattanaamornkiat W, Viriyakitja D, Monkongdee P, Sitti W, Antiretroviral therapy during tuberculosis treatment and marked reduction in death rate of HIV-infected patients, Thailand. Emerg Infect Dis. 2007;13:1001–7. 18214171 10.3201/eid1307.061506 PMC2878231

[3] Cain KP, Kanara N, Laserson KF, Vannarith C, Sameourn K, Samnang K, The epidemiology of HIV-associated tuberculosis in rural Cambodia. Int J Tuberc Lung Dis. 2007;11:1008–13. 17705980

[4] Quy HT, Cobelens FG, Lan NT, Buu TN, Lambregts CS, Borgdorff MW. Treatment outcomes by drug resistance and HIV status among tuberculosis patients in Ho Chi Minh City, Vietnam. Int J Tuberc Lung Dis. 2006;10:45–51. 16466036

[5] Thuy TT, Shah NS, Mai HA, Do TN, Duong T, Truong L, HIV-associated TB in An Giang Province, Vietnam, 2001–2004: epidemiology and TB treatment outcomes. PLoS One. 2007;2:e507. 10.1371/journal.pone.0000507 17551587 PMC1876817

[6] Heller T, Cain KP, Neal J, Daravuth E, Vannarith C, Hersh B. Tuberculosis as a late complication of HIV infection in Banteay Meanchey, Cambodia. Union World Conference against Lung Disease; 2006 Oct 31–Nov 4; Paris, France; 2006.

[7] Manosuthi W, Chottanapand S, Thongyen S, Chaovavanich A, Sungkanuparph S. Survival rate and risk factors of mortality among HIV/tuberculosis-coinfected patients with and without antiretroviral therapy. J Acquir Immune Defic Syndr. 2006;43:42–6. 10.1097/01.qai.0000230521.86964.86 16885778

[8] Nissapatorn V, Kuppusamy I, Sim BL, Quek KF, Khairul Anuar A. Tuberculosis in HIV/AIDS patients: a Malaysian experience. Southeast Asian J Trop Med Public Health. 2005;36:946–53. 16295550

[9] Varma JK, Wiriyakitjar D, Nateniyom S, Anuwatnonthakate A, Monkongdee P, Sumnapan S, Evaluating the potential impact of the new Global Plan to Stop TB: Thailand, 2004-2005. Bull World Health Organ. 2007;85:586–92. 10.2471/BLT.06.038067 17768516 PMC2636378

[10] Ansari NA, Kombe AH, Kenyon TA, Hone NM, Tappero JW, Nyirenda ST, Pathology and causes of death in a group of 128 predominantly HIV-positive patients in Botswana, 1997–1998. Int J Tuberc Lung Dis. 2002;6:55–63. 11931402

[11] Lucas SB, Hounnou A, Peacock C, Beaumel A, Djomand G, N'Gbichi JM, The mortality and pathology of HIV infection in a west African city. AIDS. 1993;7:1569–79. 10.1097/00002030-199312000-00005 7904450

[12] Lucas SB, Odida M, Wabinga H. The pathology of severe morbidity and mortality caused by HIV infection in Africa. AIDS. 1991;5(Suppl 1):S143–8. 1669911

[13] Rana FS, Hawken MP, Mwachari C, Bhatt SM, Abdullah F, Ng'ang'a LW, et al. Autopsy study of HIV-1-positive and HIV-1-negative adult medical patients in Nairobi, Kenya. J Acquir Immune Defic Syndr. 2000;24:23–9. 10877491 10.1097/00126334-200005010-00004

[14] Mermin J, Ekwaru JP, Liechty CA, Were W, Downing R, Ransom R, Effect of co-trimoxazole prophylaxis, antiretroviral therapy, and insecticide-treated bednets on the frequency of malaria in HIV-1-infected adults in Uganda: a prospective cohort study. Lancet. 2006;367:1256–61. 10.1016/S0140-6736(06)68541-3 16631881

[15] World Health Organization, Roll Back Malaria, and United Nations Children’s Fund. World malaria report 2005. Geneva: The Organization and The Fund; 2005 [cited 2008 Mar 10]. Available from http://www.rollbackmalaria.org/wmr2005/pdf/WMReport_lr.pdf

[16] Sanguanwongse N, Cain KP, Suriya P, Nateniyom S, Yamada N, Wattanaamornkiat W, Antiretroviral therapy for HIV-infected tuberculosis patients saves lives but needs to be used more frequently in Thailand. J Acquir Immune Defic Syndr. 2008;48:181–9. 10.1097/QAI.0b013e318177594e 18520676

[17] Mukadi YD, Maher D, Harries A. Tuberculosis case fatality rates in high HIV prevalence populations in sub-Saharan Africa. AIDS. 2001;15:143–52. 10.1097/00002030-200101260-00002 11216921

[18] Kalou M, Sassan-Morokro M, Abouya L, Bile C, Maurice C, Maran M, Changes in HIV RNA viral load, CD4+ T-cell counts, and levels of immune activation markers associated with anti-tuberculosis therapy and cotrimoxazole prophylaxis among HIV-infected tuberculosis patients in Abidjan, Cote d'Ivoire. J Med Virol. 2005;75:202–8. 10.1002/jmv.20257 15602734

[19] Morris L, Martin DJ, Bredell H, Nyoka SN, Sacks L, Pendle S, Human immunodeficiency virus-1 RNA levels and CD4 lymphocyte counts, during treatment for active tuberculosis, in South African patients. J Infect Dis. 2003;187:1967–71. 10.1086/375346 12792875

[20] Boeree MJ, Sauvageot D, Banda HT, Harries AD, Zijlstra EE. Efficacy and safety of two dosages of cotrimoxazole as preventive treatment for HIV-infected Malawian adults with new smear-positive tuberculosis. Trop Med Int Health. 2005;10:723–33. 10.1111/j.1365-3156.2005.01433.x 16045458

[21] Grimwade K, Sturm AW, Nunn AJ, Mbatha D, Zungu D, Gilks CF. Effectiveness of cotrimoxazole prophylaxis on mortality in adults with tuberculosis in rural South Africa. AIDS. 2005;19:163–8. 10.1097/00002030-200501280-00008 15668541

[22] Mwaungulu FB, Floyd S, Crampin AC, Kasimba S, Malema S, Kanyongoloka H, Cotrimoxazole prophylaxis reduces mortality in human immunodeficiency virus-positive tuberculosis patients in Karonga District, Malawi. Bull World Health Organ. 2004;82:354–63. 15298226 PMC2622842

[23] Wiktor SZ, Sassan-Morokro M, Grant AD, Abouya L, Karon JM, Maurice C, Efficacy of trimethoprim-sulphamethoxazole prophylaxis to decrease morbidity and mortality in HIV-1-infected patients with tuberculosis in Abidjan, Côte d’Ivoire: a randomised controlled trial. Lancet. 1999;353:1469–75. 10.1016/S0140-6736(99)03465-0 10232312

[24] Centers for Disease Control and Prevention. Spotlight on Syndemics [cited 2007 May 5]. Available from http://www.cdc.gov/syndemics/overview.htm

[25] World Health Organization. Global tuberculosis control: surveillance, planning, financing. WHO report 2007. Geneva: The Organization; 2007. WHO/HTM/TB/2007.376 [cited 2008 Jun 22]. Available from http://www.who.int/tb/publications/global_report/2007/en/index.html

[26] Nateniyom S, Jittimanee SX, Viriyakitjar D, Jittimanee S, Keophaithool S, Varma JK. Provider-initiated diagnostic HIV counseling and testing in tuberculosis clinics in Thailand. Int J Tuberc Lung Dis. 2008;12:955–61. 18647457

[27] Baiden F, Bawah A, Biai S, Binka F, Boerma T, Byass P, Setting international standards for verbal autopsy. Bull World Health Organ. 2007;85:570–1. 10.2471/BLT.07.043745 17768508 PMC2636382

[28] Chandramohan D, Maude GH, Rodrigues LC, Hayes RJ. Verbal autopsies for adult deaths: their development and validation in a multicentre study. Trop Med Int Health. 1998;3:436–46. 9657505 10.1046/j.1365-3156.1998.00255.x

[29] Hosegood V, Vanneste AM, Timaeus IM. Levels and causes of adult mortality in rural South Africa: the impact of AIDS. AIDS. 2004;18:663–71. 10.1097/00002030-200403050-00011 15090772

[30] Lopman BA, Barnabas RV, Boerma JT, Chawira G, Gaitskell K, Harrop T, Creating and validating an algorithm to measure AIDS mortality in the adult population using verbal autopsy. PLoS Med. 2006;3:e312. 10.1371/journal.pmed.0030312 16881730 PMC1526767

[31] Lulu K, Berhane Y. The use of simplified verbal autopsy in identifying causes of adult death in a predominantly rural population in Ethiopia. BMC Public Health. 2005;5:58. 10.1186/1471-2458-5-58 15935096 PMC1164421

[32] Setel PW, Whiting DR, Hemed Y, Chandramohan D, Wolfson LJ, Alberti KG, Validity of verbal autopsy procedures for determining cause of death in Tanzania. Trop Med Int Health. 2006;11:681–96. 10.1111/j.1365-3156.2006.01603.x 16640621

[33] Sharma SK, Kadhiravan T, Banga A, Goyal T, Bhatia I, Saha PK. Spectrum of clinical disease in a series of 135 hospitalised HIV-infected patients from north India. BMC Infect Dis. 2004;4:52. 10.1186/1471-2334-4-52 15555069 PMC535567

[34] Thwaites GE, Duc Bang N, Huy Dung N, Thi Quy H, Thi Tuong Oanh D, Thi Cam Thoa N, The influence of HIV infection on clinical presentation, response to treatment, and outcome in adults with tuberculous meningitis. J Infect Dis. 2005;192:2134–41. 10.1086/498220 16288379

[35] World Health Organization. The global MDR-TB and XDR-TB response plan. Geneva: The Organization; 2007. WHO/HTM/TB/2007.387 [cited 2008 Jun 22]. Available from http://whqlibdoc.who.int/hq/2007/WHO_HTM_TB_2007.387_eng.pdf.

[36] Burman W, Benator D, Vernon A, Khan A, Jones B, Silva C, Acquired rifamycin resistance with twice-weekly treatment of HIV-related tuberculosis. Am J Respir Crit Care Med. 2006;173:350–6. 10.1164/rccm.200503-417OC 16109981

[37] Dean GL, Edwards SG, Ives NJ, Matthews G, Fox EF, Navaratne L, Treatment of tuberculosis in HIV-infected persons in the era of highly active antiretroviral therapy. AIDS. 2002;16:75–83. 10.1097/00002030-200201040-00010 11741165

[38] Dheda K, Lampe FC, Johnson MA, Lipman MC. Outcome of HIV-associated tuberculosis in the era of highly active antiretroviral therapy. J Infect Dis. 2004;190:1670–6. 10.1086/424676 15478074

[39] Blanc FX, Havlir DV, Onyebujoh PC, Thim S, Goldfeld AE, Delfraissy JF. Treatment strategies for HIV-infected patients with tuberculosis: ongoing and planned clinical trials. J Infect Dis. 2007;196(Suppl 1):S46–51. 10.1086/518658 17624825

[40] Srisuwanvilai LO, Monkongdee P, Podewils LJ, Ngamlert K, Pobkeeree V, Puripokai P, Performance of the BACTEC MGIT 960 compared to solid media for detection of *Mycobacterium* in Bangkok, Thailand. Diagn Microbiol Infect Dis. 2008;61:402–7. 10.1016/j.diagmicrobio.2008.02.015 18440177

